# How intercultural experience affects university students’ gender views: potential for transforming higher education in Japan

**DOI:** 10.1007/s12564-022-09801-5

**Published:** 2022-11-28

**Authors:** Kiyoko Uematsu‑Ervasti, Kumiko Kawachi

**Affiliations:** 1 Extension School, University of Oulu, Oulu, Finland; 2 International Strategy Organization, Yokohama National University, Yokohama, Japan

**Keywords:** Gender and education, Internationalization on campus, Transformative learning, Japanese higher education, The BEVI, Sustainable development goals

## Abstract

Reducing gender disparities in Japan is an urgent issue that requires the attention of multilevel stakeholders, including higher education institutions. Under Sustainable Development Goal 4, target 4.7 calls for educational institutions, including universities, to explore innovative approaches to tackle issues such as gender inequality. The transition from student life to adulthood is a crucial time for university students as they face and become aware of gender inequalities in society. This study examines various opportunities in higher education, such as short study-abroad programs and globalization-related coursework, that positively influence university students’ understanding of others and potentially broaden their gender perspectives. Historically, the impacts of studying abroad and intercultural experiences have only been subjectively assessed based on participants’ self-evaluation. However, by using the Beliefs, Events, and Values Inventory, quantified data can be used to evaluate the nature and form of depth-based growth and compare three cases for potential changes before, after, or during educational programs. The study’s findings shed light on opportunities and barriers to transforming students’ views especially on gender issues. The study reveals (1) signs of improvement in students’ gender perspectives after participating in a short study-abroad program, (2) that international students with diverse experiences tend to demonstrate lower gender traditionalism, and (3) that freshmen and sophomores have relatively moderate views on gender norms, indicating that further intervention could improve their gender perspectives. The results showcase the possibility of developing meaningful study-abroad programs and other coursework to drive the positive change in students’ perspectives, especially regarding gender.

## Introduction

The Japanese society has lagged behind the international community in promoting gender equality, which has negatively affected the evaluation of Japan's efforts to achieve the sustainable development goals (SDGs). In the *Sustainable Development Report 2021*, Japan was ranked 18th among the 165 ranked countries (Sachs et al., 2021); however, the report pointed out that Japan needs significant improvements in the field of gender equality and women’s empowerment. In the *Global Gender Gap Report 2021* released by the World Economic Forum, Japan ranked 120th out of 156 countries (World Economic Forum, 2021). The Global Gender Gap is an index for measuring the degree of gender inequality based on four key dimensions (economic participation and opportunity, educational attainment, health and survival, and political empowerment). Japan has inched up only one place, from 121st in the previous report. With this result, Japan is ranked the lowest among G7 countries and the lowest among East Asian countries, particularly in the political and economic fields. In addition to these major challenges, researchers also pointed out the gender gap in the field of higher education. Compared with other Organization for Economic Co-operation and Development (OECD) countries, Japan has been lagging behind the international trend of gender mainstreaming in higher education (Ito, 2016).

Higher education institutions are expected to play a significant role in promoting the transformation of the Japanese society. Ikemoto (2018) pointed out that one of the reasons for the low enrollment rate of Japanese female students in higher education relates to the low employment rate of females with higher education. Obtaining a higher degree in Japan has not guaranteed women better economic opportunities. Under these circumstances, Ikemoto (2018) stressed that it is essential to pay more attention to higher education from the perspectives of promoting women’s social advancement, including diversifying educational contents and the way of learning (Ikemoto, 2018). SDG Goal 4.7 also calls for educational institutions, including higher education, to play a key role in exploring innovative approaches to tackle issues, such as gender inequality.

Globalization has accelerated the internationalization of higher education in many countries (Altbach & Knight, 2007). From the mid-1980s to the mid-1990s, the number of Japanese students studying abroad has rapidly increased, especially in the United States (U.S.). In the late 1990s, the number of Japanese women coming to the U.S. as international students was almost equal to, or in some years, slightly higher than that of men. Both Japanese female and male students chose to study abroad for some similar positive reasons, such as improving English, having the U.S. study experience broadening their views on international and intercultural issues, and building networks (Ono & Piper, 2004). As shown in a study by Ono and Piper (2004), there was no significant difference in terms of the number of Japanese males and females studying in the U.S.; however, Williams (2020) noted that the overwhelming number of studies regarding Japanese youth living abroad have focused on the Japanese women and not men.

Regarding the internationalization of higher education in Japan, it has changed dramatically in the past decade. According to Kobayashi (2017), the number of Japanese people studying abroad for a long-term period peaked in 2004 (82,945 individuals) and declined in 2014 (53,197 individuals; p. 65). On the other hand, over the past five years, the number of international students who come to Japan has rapidly increased from 184,155 in 2014 to 312,214 in 2019 (Japan Student Services Organization, 2022). One prominent characteristic of study-abroad programs among Japanese students is the increase of “super-short-term study-abroad” programs that last from one week to less than one month. In 2008, the Japanese government increased the funding for the scholarship scheme by the Japan Student Services Organization (JASSO), thereby accelerating the development of short study-abroad programs. Shimmi and Ota (2018) noted that the number of students who have studied abroad in this “super-short-term” study-abroad style tripled between 2009 and 2016 (p. 13).

Kitano (2020) also mentioned that 70% of Japanese students participating in study-abroad programs in recent years have chosen to study abroad for less than 1 month (pp. 185–186). Clearly, the majority of Japanese college students have chosen this shorter program. The increase in the number of students who prefer this form of studying abroad is due to the environment surrounding Japanese university students. Although students desire to experience living and studying abroad, they do not prefer studying abroad for a more extended period because of time constraints with job hunting, national exams, club activities, as well as the burden of high financial costs (Kitano, 2020; Kobayashi, 2017; Shimmi & Ota, 2018). The prevalence of short-term study-abroad programs at Japanese universities gives the opportunity to those students who had given up on studying abroad for long-term due to the aforementioned reasons.

This study explores the possibility of various opportunities in higher education, such as short study-abroad programs and globalization-related coursework to positively impact university students’ understanding of others and potentially lead to broadening their gender perspectives. This study compares three cases where the beliefs, events, and values inventory (BEVI) was used to analyze emotional and psychological changes before, after, or during educational programs. The three cases involved groups of Japanese students: (1) who took part in a short-term study-abroad program in the Republic of the Philippines, (2) who took a course related to gender, and (3) who registered for a course related to globalization. All the data come from students studying at the Yokohama National University (hereinafter YNU) in Kanagawa. The results showcase the possibility of developing meaningful study-abroad programs as well as other intercultural learning opportunities to trigger a positive change in students’ perspectives, especially regarding gender.

## Literature review

### Gender imbalances in the Japanese society

Looking at the recent volumes of the *Global Gender Gap Report*, Japan has been characterized by a remarkably low ranking in the political and economic fields. There is also a great concern in the field of education. In Japan, there is a notable gap in the ratio of male to female students, especially in masters and doctoral programs. According to *Japanese Science and Technology Indicators* (2021), the students enrolled in masters programs in the 2020 academic year comprised 50,000 males and 22,000 females. As with masters programs, the number of female students enrolled in doctoral programs in Japan was less than half of that of male students (p. 105).

There is also a huge gap in Japanese universities in terms of the ratio of male and female faculty members. Miyake (2018) pointed out the “lack of gender mainstreaming accountability” in Japanese universities; this is evident in how Japanese universities are organized and operated, with males accounting for 80% of administrative positions. Miyake (2018) also noted that increasing the number of female faculty members in universities is vital because female professors could become role models for their female students and it leads to diversity in the way of imparting academic knowledge (pp. 55–56). 

The areas of economics and politics, specifically, are the areas where there is a significant gender gap. Looking at the latest *Global Gender Gap Report,* the percentage of female legislators, senior officials, and managers is just 14.7% (139th place). In politics, female politicians in the Japanese parliament account for only 9.9% of the total (ranking 147th (World Economic Forum, 2021, pp. 233–234)). These results show that Japan lags far behind in the appointment of female leaders in both the economic and political spheres. The phenomenon of female under-representation in Japanese leadership begins after female students graduate from college. According to Sakata (2019), who surveyed 200 men and 200 women to investigate their leadership experiences, there was no difference between males and females in terms of leadership experiences during their elementary school through college years. However, after entering the workforce, there is a change in the number of leadership experiences among the two groups: more males are appointed to leadership positions than females. This result indicates that females in Japan have fewer opportunities to take on leadership roles, such as managerial positions, after entering the workforce. In other words, the college years are a time of great transition for Japanese youths. Some studies have shown that learning environments during college play a role in constructing students’ perspectives on gender inequality. Bryant (2003) pointed out that when students are exposed to diverse environments during their college days, for instance, through participation in racial and ethnic awareness workshops, this may increase their awareness of social inequalities, including gender issues (p. 138). ​​To unfold the potential role of universities in addressing social inequality through their work in higher education, study-abroad programs are discussed as an approach to broaden students’ perspectives on gender.

### Study-abroad programs and gender education in universities

The significance of study-abroad programs as a means to promote internationalization in higher education is that it allows students to “improve their language skills that can only be obtained overseas and acquire specialized knowledge” (Sugimura, 2020). In addition to these main objectives, students can gain insights into local people’s lives and culture, which may not be possible when living in their home country. A benefit of studying abroad is that it gives students the opportunity to re-examine their own cultural values and social systems (Jessup-Anger, 2008).

Research that focuses on students’ views on gender in the context of studying abroad has continued since the 1990s, although the number remains limited. There is a common trend among the previous studies; they are mostly case studies targeted for short study-abroad programs conducted by Western universities, and most of the participating students are Caucasian (e.g., Jessup-Anger, 2008; Phillion et al., 2009; Squire et al., 2015; Talburt & Stewart, 1999; Twombly, 1995).

A short-term study-abroad program with activities related to gender issues was found to positively affect students’ understanding of their social identity, including gender (Squire et al., 2015). Based on the findings of the study, Jessup-Anger (2008) stated that comprehensive educational interventions by staff and teachers are necessary to bring educational benefits of gender understanding to study-abroad programs. The author further noted that participating students are less likely to become aware of the gender dynamics of their study-abroad destination without educational interventions (pp. 366–367). Jessup-Anger (2008) stated that comprehensive educational interventions by staff and teachers are essential to bring about the educational effect of gender understanding possible through studying abroad. As gender is socially constructed (Wharton, 2012), a short study-abroad could trigger the transformation of students’ perspectives, resulting in liberalized gender-role attitudes.

### Study-abroad and transformative learning

There have been studies indicating that studying abroad has the potential for transformative learning (e.g., Bell et al., 2016; Kumi-Yeboah, 2014; Tarrant, 2010; Vatalaro et al., 2015), where reflection and active learning allow students to gain a critical understanding of the world, others, and themselves. Being situated in an unfamiliar environment invites students to explore their position in the world and possibly challenge preconceptions that had previously guided them to think or act in certain ways.

Transformative learning is defined as learning that transforms “frames of reference,” including sets of fixed assumptions and expectations (Mezirow, 1997, 2003). Frames of reference are the structures of those assumptions, and are directly connected to how students understand their experiences. A transformative learning process can be facilitated when learners critically analyze their own frames of reference or even others’ assumptions. Such a positive step can lead to the construction of more diverse, inclusive, and self-reflective practices that empower students (Strange & Gibson, 2017). To start the transformational learning process, “we must learn to make our own interpretations rather than act on the purposes, beliefs, judgments, and feelings of others” (Mezirow, 1997, p. 5). In the context of a short study-abroad program, the transformative learning process is triggered by deep reflection and conversations in unfamiliar local contexts that are out of the students’ comfort zone (Kikuchi, 2018). Studying abroad allows students to develop an interest in current global affairs and improve their cross-cultural adaptability (e.g., Deardorff, 2014; Vande Berg et al., 2009). These experiences are also possible in a classroom learning environment when participants get exposed to people of different languages, values, and cultural backgrounds and where opportunities are presented to challenge their own values. Students are then given a chance to reconsider preconceived assumptions built within their frames of reference. Given that there are articulated learning goals for either a short study-abroad program or any other coursework focusing on globalization or intercultural themes, such programs could potentially transform or at least give students an opportunity to reflect on their conventional thinking, for example about gender issues.

There is a continuous trend and high demand for short study-abroad programs and other forms of intercultural learning, such as Collaborative Online International Learning (COIL) or bilingual collaborative classes that embrace local and international students (Bysouth & Ikeda, 2019). This presents a momentum to find the first step toward fostering “autonomous” and “independent” thinkers who are simultaneously empathic, open-minded, and willing to engage critically with the assumptions and social norms with which they are familiar as a result of reflective thinking. This study examines to what extent intercultural experiences favorably influence university students’ understanding of others and the opportunities in universities to positively influence students’ frame of reference, specifically by broadening their gender perspective.

## Research design and method

This study examines to what extent intercultural experiences positively impact university students’ understanding of others and their potential to influence students’ gender perspectives. Moreover, this study sheds light on how assumptions can be shifted or transformed, thereby broadening students’ gender perspectives. To investigate Japanese university students’ gender perspectives, the psychological test called beliefs, events, and values inventory (BEVI) was used to determine students’ emotional and psychological state. To supplement the quantitative data from the BEVI instrument and explore the meanings represented in statistics, we used qualitative data from either follow-up semi-structured interviews, the responses collected to open questions on the BEVI, or interviews with the course instructor to gain insights. Data from three different cases where student groups have different intercultural experiences and backgrounds were compared. Drawing on these three cases, this study demonstrates patterns that are crucial in understanding students’ views on others and, more specifically, the gender perspectives among university students.

### The BEVI instrument for measuring the impact of studying abroad and intercultural experiences

The BEVI is a comprehensive, mixed-method, and psychometric instrument based on grounded theory, developed in the 1990s by Dr. Craig N. Shealy, a clinical psychologist, for measuring a wide range of learning, growth, and development processes and outcomes, including the educational effects of studying abroad (See www.thebevi.com). The instrument was developed over 30 years ago by psychologists, test theory experts, and psychometricians, including Japanese and Chinese experts and is very different from an opinion survey or questionnaire. By adopting this approach, Professor Shealy and his team derived scale scores by comparing each item’s response to approximately 10,000 standardized data from around the world (Nishitani, 2020). Among other constructs, the BEVI can measure a wide range of psychological, critical thinking, understanding of others, and cross-cultural acceptance structures based on the Equilintegration (EI) Theory and the EI Self (Nishitani, 2018; Shealy, 2016). Historically, the impacts of studying abroad and intercultural experiences have mostly been assessed based on participants’ self-evaluation. However, by using the BEVI, quantified data can be used to evaluate the nature and form of depth-based changes that occur at the individual, group, course, and possibly the institutional levels over the duration of a program or an intervention, including study-abroad programs (Nishitani, 2018). One of the unique features of the BEVI is its ability to quantify changes in students along a 17-scale, two-time frame measure: T1 and T2 (before and after), which are measured at the beginning and end of the targeted program. Before participating in this study, respondents were presented with a consent form to take part in the survey for the BEVI program. The consent form comprised details regarding the use of the result, including the rule that the results will be handled only as a “group” and individual results will be inaccessible for the administrators of the BEVI. The psychological test was administered only after consent was received. Based on the collected data, group reports were created, and the aggregate profiles were used to compare patterns within and between the cases.

The BEVI survey contains 185 questions regarding beliefs, values, and worldviews. Of the 17 scales, there are three scales under the BEVI domain of “Other Access,” which includes the measurement of religious traditionalism, gender traditionalism, and sociocultural awareness (Other Access, Scale 13–15).

This study focuses on “gender traditionalism” (Scale 14). The scale is designed to measure how one perceives and experiences traditional/basic views of gender and gender roles, based on a range of interrelated beliefs, for example, if one believes that men and women are “built” to be a certain way or one prefers traditional or simple views of gender and gender roles (Wandschneider et al., 2016). A higher score on gender traditionalism indicates that there is a stronger tendency to view gender in a fixed way, whereas a lower score means that concepts of gender behaviors and roles are more flexible.

Among the data recorded across 17 scales in the BEVI report, this study focuses on the data collected on gender traditionalism among students in three different cases. From the BEVI’s standpoint, it is always important to focus on within-group variability, which may be considerable across different settings and contexts (Shealy, 2016). Keeping this point in mind, according to some previous BEVI-related studies that focus on college students, gender traditionalism among male students in Japanese universities is not particularly high compared with students in other countries. For instance, among students who took a multicultural undergraduate course at a midwestern university in the United States, politically conservative and religious males tended to have higher scores on the scale of gender traditionalism. In an analysis by gender at the beginning of the course (T1), male students had much higher scores than females: males were the 56th and females were the 20th percentile (Iseminger et al., 2020). Regarding first-year students in Japanese universities, an example from Hiroshima University (*N* = 19) showed that the overall average rate was in the 32nd percentile for gender traditionalism. However, male students had higher scores (58th percentile) at the beginning of the study-abroad program, whereas female students had relatively lower scores (26th percentile; i.e., males had more than twice the scores of females on this scale; Nishitani, 2020).

### Student groups: three cases

#### Case 1: a short-term study-abroad program and its participants

The short-term study-abroad program labeled an “overseas intensive camp,” at YNU is designed to provide opportunities to acquire English language proficiency while taking language training at partner universities. These short-term programs are often recognized as the first step for students to experience studying abroad, often leading them to consider applying for a long-term study-abroad program after the trial experience. Most of the participants are Japanese nationals studying in undergraduate programs. The first case focuses on a group of students who attended a short study-abroad program in the Republic of the Philippines (hereinafter, the Philippines) which is one of the popular destinations in Asia to acquire English language proficiency.

From February 26 to March 14, 2020, seven students (three females and four males) participated in an intensive program organized at YNU’s partner university, Santo Tomas University, located in Manila. During the 18-days program, students engaged in interactive English learning, focusing on everyday English and improving conversational skills. On usual days, students had the conversational English course from 8 am to 12 pm and 1 pm to 5 pm. Then, there were two *language-on-foot* excursions. For the first excursion during the first week, students visited the library, museum, research centers, and academic units (2 h). In the second week, another excursion was scheduled to take place in the Walled City for the whole day (8 h); however, it was canceled due to the sudden increase of COVID-19 cases in the Philippines. A unique characteristic of the program was that its participants studied English intensively on the campus while living in a university dormitory located on campus, and only the weekday programs were supervised by Santo Tomas University. They also had opportunities to explore the city after the class and during the weekends. Students were free to do what they want during weekends and after 5 pm on a weekday; they made plans together and went sightseeing to distant cities. Further, their activities outside the campus were limited toward the end of the program due to the curfew. They took the first BEVI test (T1) before arriving in the Philippines and took the second BEVI test (T2) after returning to Yokohama. The results are discussed in Sect. 4.

#### Case 2: an international undergraduate program and its participants

At YNU, there is an English-taught undergraduate program called Yokohama Creative City Studies (YCCS) program, which focuses on the following three areas, namely (1) Japanese language and cultural skills, (2) Global common competencies, and (3) Specialized competencies in collaboration, leadership, and facilitation (YCCS, 2021a). The eligibility to apply for the YCCS program (as of 2020) includes having completed a 12-year curriculum in an educational institution abroad (YCCS, 2021b), which means that all the applicants had completed their basic schooling abroad. Many of the YCCS students have diverse linguistic and cultural backgrounds and some have experience living in multiple countries.

During autumn, a course called “Gender and an Inclusive Society” is offered to students in the YCCS program. The purpose of the course is “…to become aware of gender issues and their complex intersections with age, class, and ethnicity through class discussions, readings, and by doing mini fieldwork" (YNU, 2021a). Eleven students (seven females and four males) participated in 2020, and they were a diverse group with different racial, ethnic, cultural as well as educational backgrounds, contributing to a global learning environment. Their backgrounds included East Asia, Southeast Asia, South America, North America, and North Europe, and some students had multicultural backgrounds. Many students had lived and received education in different countries. The series of lectures covered topics on ethnic minority groups in Japanese and Latin American societies. One of the main themes in the course was social inequality, and different types of social groups were discussed, including gender issues. A research-oriented approach was applied. For instance, one of the assignments required the students to discover social issues related to gender and ethnicity, conduct their own interviews, analyze them, and write a report based on their findings. The course was provided using the Zoom meeting system, and the tests were taken at the beginning (T1) and at the end (T2) of the course.

#### Case 3: an intercultural communication course and its participants

The final case involved a large group of students who took a course called “*Gurobaruka to nihonjin*” (translated to “Japanese Ways as Intercultural Communication”) at YNU during autumn. This course contains various lectures on cross-cultural communication, global human resources, and collaboration in international business settings. The course’s learning outcome is set so that “students will be able to play an active role globally in the future and establish and practice their own way of cross-cultural communication” (YNU, 2021b).^1^

There were 10 respondents in this case (five females and five males). This course tends to attract first and second year students who are interested in intercultural issues or a global career in the future. The T1 data were collected at the beginning of the course and T2 data were collected toward the end of the course. Here is a summary and overview of the participants and respondents from the three cases (Table 1).

**Table 1:** Overview of the three cases

	Number of participants, (Female, Male)	Number of respondents (Female, Male)	Program period (duration)	Timing of T1	Timing of T2	Demographics
Case 1: short-term exchange	7 (*F* = 3, *M* = 4)	7 (*F* = 3, *M* = 4)	February 2020–March 2020 (18 days)	January 2020	April 2020	All Japanese students
Case 2: Gender Inclusive Society Course	11 (*F* = 7, *M* = 4)	11 (*F* = 7, *M* = 4)	October 2020–February 2021(90 min, 15 classes)	October 2020	March 2021	Multicultural backgrounds
Case 3: Japanese Ways as Intercultural Communication Course	18 (*F* = 9, *M* = 9)	10 (*F* = 5, *M* = 5)	October 2021–February 2022(90 min, 15 classes)	October 2021	February 2022	All Japanese students

All of the three cases contain T1 and T2 test results and all the tests are taken by students at YNU during the period of January 2020–February 2022

## Findings

The following presents a summary of each case with regard to students’ understanding of others. Based on the BEVI test scores, the group results are discussed in terms of average scores. The BEVI allows analysis by subgroup to gain a deeper understanding of the overall data. In this study, the aggregate reports by gender are presented to further analyze the scores indicating gender differences. Normally, the higher the number, the higher the score is on the respective scale. However, for items such as gender traditionalism, lower numbers indicate better results, as lower scores on these scales mean that the participants’ thinking is not as fixed on conventional ideas or bound by traditionalism. Here is an overview of the data on gender traditionalism and the discussion case by case (Table 2).

**Table 2:** Summary of T1 and T2 scores on gender traditionalism by gender

	Male			Female		
	T1	T2	Change	T1	T2	Change
Case 1: short-term exchange	46	39	7 percentile decrease ↓	21	7	14 percentile decrease ↓
Case 2: GENDER Inclusive Society Course	12	16	4 percentile increase	20	16	4 percentile decrease
Case 3: Japanese Ways as Intercultural Communication Course	59	47	12 percentile decrease ↓	49	46	3 percentile decrease

The bold texts indicate statistical significance (more than the 5th percentile), and the arrow shows the direction for the significant change. Those changes that showed less than the 5th percentile are not statistically significant

### Case 1: the short-term study-abroad program (T1/T2 report)

**Fig. 1:**
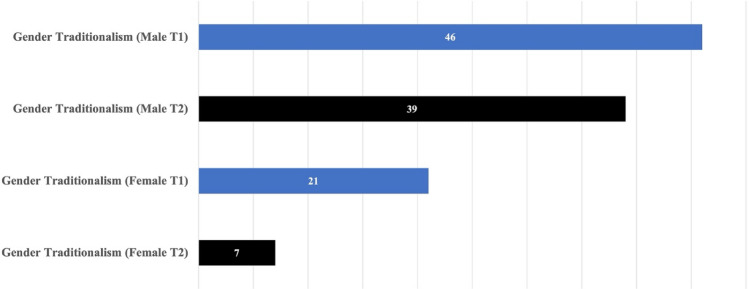
Case 1: T1/T2 data on gender traditionalism by gender

Students who attended the short-term study-abroad program (*N* = 7) showed significant changes in terms of gender traditionalism.^2^ Analysis of the data by gender showed significant differences in the amount and direction of the changes between male and female students for gender traditionalism (See Fig. 1).

Figure 1 shows a 7 percentile decrease among male students (T1: 46, T2: 39), indicating that male students (*N* = 4) in the program exhibited a moderate level of gender traditionalism before the program. After returning from the study-abroad program, an improvement (by 7 percentile) is observed in their gender views. For female students (*N* = 3) who exhibited a lower level of gender traditionalism at the beginning of the program, the results similarly show an improvement (by 14 percentile; T1: 21, T2: 7). It is generally understood that young women tend to demonstrate more egalitarian gender-role attitudes than young men (Fan & Marini, 2000). Moreover, evidence from various educational institutions that employ the BEVI has demonstrated that scores of gender traditionalism are lower among female students (e.g., Iseminger et al., 2020; Nishitani, 2020; Toya & Toma, 2019).

Follow-up interviews were conducted to examine aspects that may have contributed to the positive change in the students' gender perspectives in this program (Kawachi & Uematsu-Ervasti, 2021). The preliminary qualitative study revealed that during the 18-day stay in the Philippines, students observed and engaged with rather empowered, independent, and active female roles. For example, a male student shared his experience when encountering a female executive who was the director of the short study-abroad program. He recalled that it gave him a “very fresh impression,” as he felt that it is so rare to see a female executive in a management position in Japanese universities (Kawachi & Uematsu-Ervasti, 2021). Another male student shared, “I thought Japan was behind (in gender equality), but the feeling got even stronger” after visiting the Philippines (Kawachi & Uematsu-Ervasti, 2021). Being rather critical of the ideal image of a Japanese man who works hard but does not care about his family, he saw many family members playing together, suggesting that child-raising is not only the responsibility of women.

A female student mentioned observing some economically independent women who were also raising small children, which is very different from the traditional roles of women in Japan that she is accustomed to. She shared her perception that “women are in a weak position in Japan and cannot return to work once they quit their jobs [to get married or give birth]” (Kawachi & Uematsu-Ervasti, 2021). Other students also shared their impression that Filipino communities seem to accept diversity and are open to a flexible view of gender. In Japan, “only superwomen who work as much as men while raising children are sometimes considered as role models,” (Sakata, 2019, p. 55). However, for women’s self-affirmation, finding “appropriate” role models is beneficial. In other words, it is important to have diversity in the role models of working women (Sakata, 2019). A short-term study-abroad program allows Japanese students the exposure to a variety of role models.

The positive change regarding gender traditionalism was a rather unexpected positive effect, as it was not included in the program’s learning goals; however, the result suggests the possibility for higher education to provide students with opportunities to examine their own assumptions and broaden their frame of mind in a study-abroad program.

### Case 2: the international undergraduate program (T1/T2 report)

**Fig. 2:**
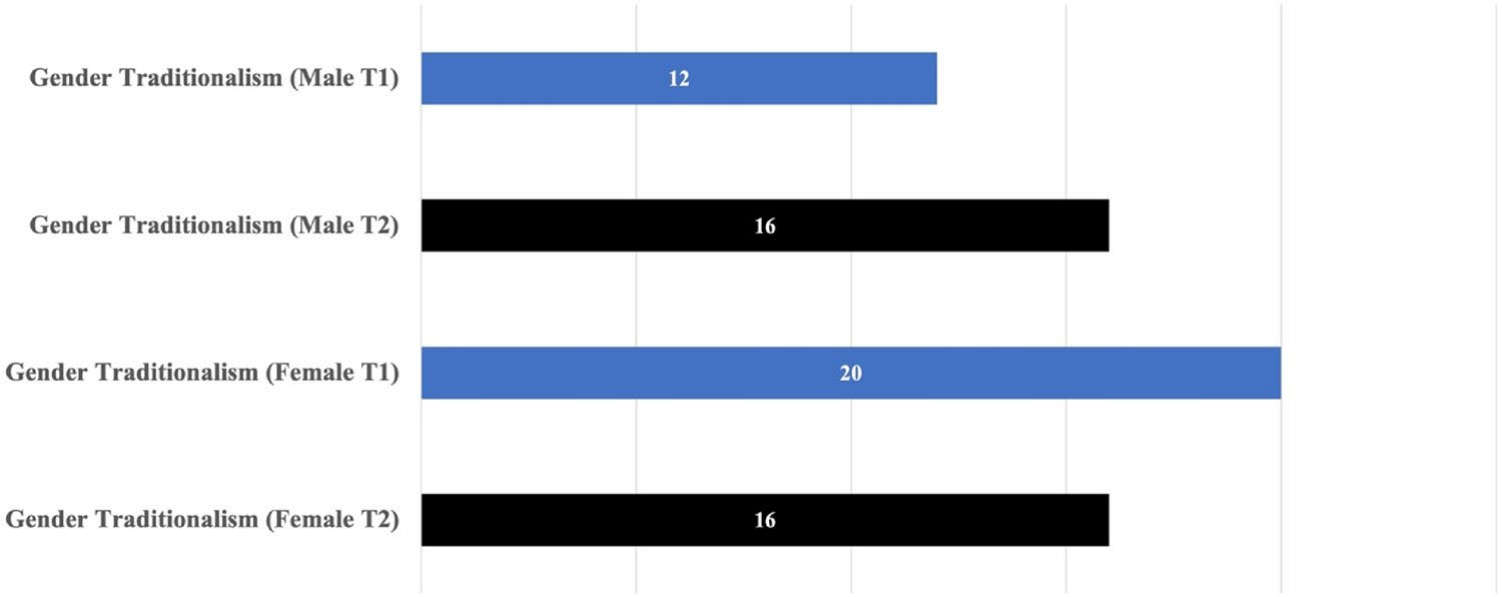
Case 2: T1/T2 data on gender traditionalism by gender

The results of Case 2, involving a student group (*N* = 11) that was part of an international undergraduate student program at YNU, showed no significant differences in the T1/T2 report on gender traditionalism, even by gender subgroup. The T1/T2 scores of male students (*N* = 4) were 12 (T1) and 16 (T2)—a 4 percentile increase, and of female students (N = 7) were 20 (T1) and 16 (T2)—a 4 percentile decrease is less than 5th percentile; therefore, it is considered that there was no statistically significant change (see Fig. 2).

However, it is important to highlight that the average scores of both females and males in Case 2 are much lower to begin with, compared with the Case 1 results. Particularly for male students, the T1 score in Case 2 is 12, which is almost one-fourth of the T1 score in Case 1 (i.e., 46). The starting scores differed substantially, and from the course instructor’s perspective, a possible explanation is that students participating in the YCCS come from diverse backgrounds, and their past experiences may have led to their diverse gender views. Not only differences in culture but also in language may have led them to challenge their frame of reference as they engaged with people of different backgrounds. Some of the recent reports using BEVI indicate that intercultural and transformative learning experiences lead students to acquire cross-cultural operational capabilities and constantly challenge their assumptions and values (Bysouth & Ikeda, 2019). It is also possible that these international students may have experienced different types of discrimination that led them to reflect critically and react strongly on topics related to social justice and power.

In addition, this course specifically focused on gender and an inclusive society. Thus, it is likely that a good number of the course participants have great academic interest in understanding diverse social groups and gender equality. These experiences may have formed their non-traditional gender views even before taking the course. It is noteworthy that although female students generally tend to have a more liberal understanding of gender than male students, this dynamic was not observed in this group. At T1, the male group scored 12, whereas the female group scored slightly higher (i.e., 16). It is commonly recognized that gender inequity is associated with women who are “on the receiving end of patriarchal imbalances” (Pendleton et al., 2016); however, male students in this course tended to address gender-related issues by highlighting some of the norms that did not necessarily receive attention, which tended to be about women (e.g., the *expected* male role of *masculine* men in society).

For Case 2, many students responded to the BEVI’s qualitative question: “Describe which aspect of this experience has had the greatest impact on you and why?” Participants shared similar sentiments in terms of developing their intercultural skills. A response that exemplifies this group is “I believe that my experience of living in different countries itself had the greatest impact on my life…I interacted with a wide variety of people from different ethnicities, ages, sex, and so on,” suggesting that experiences abroad have led to the widening of their perspectives. Another student described that “Having contact with people from different countries, cultures, and backgrounds made me think about what I want to pursue in life. I think it is beautiful how at the same time we are different, we are so similar.” This quote indicates how the students appreciate interacting with people different from themselves and value diversity.

### Case 3: the intercultural communication course (T1/T2 report)

**Fig. 3:**
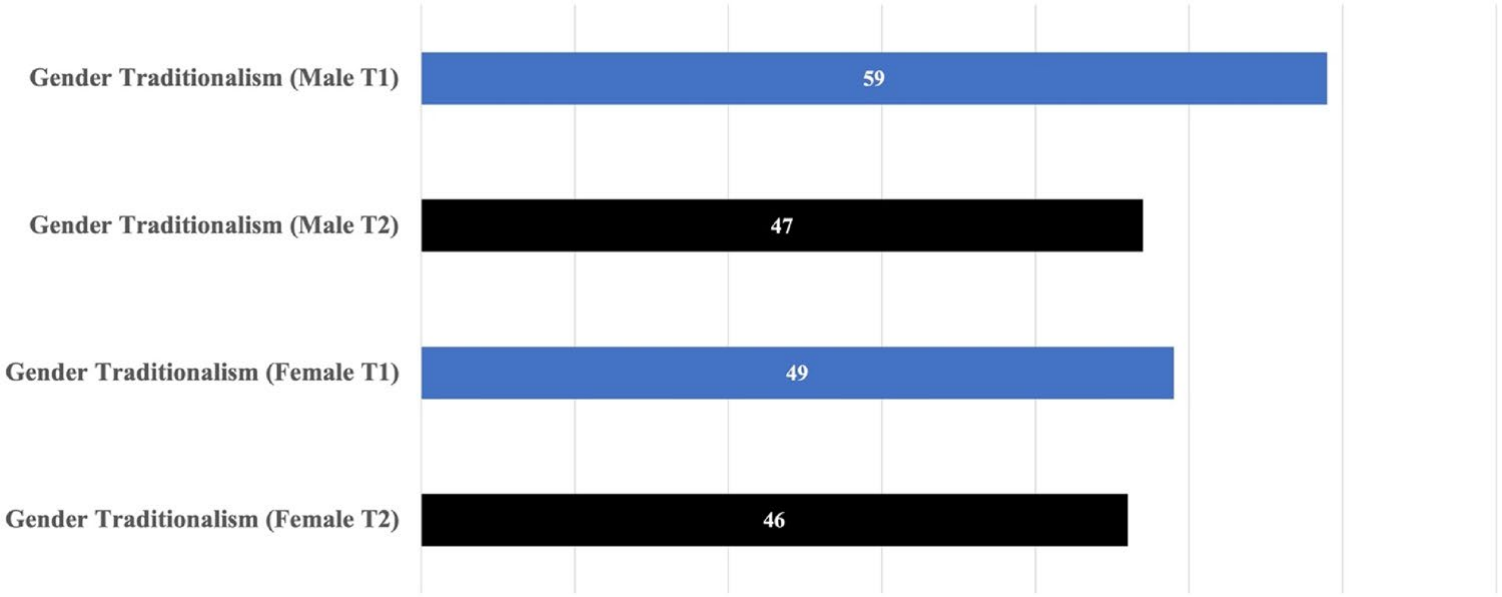
Case 3: T1/T2 data on gender traditionalism by gender

Case 3 involved Japanese students (*n* = 10) who were studying locally at YNU and those who registered for a subject called *Gurobaruka to nihonjin* (“Japanese Ways as Intercultural Communication”) during the 2021 Autumn semester. As Fig. 3 shows, the T1 score on gender traditionalism among males is 59 and females is 49. The statistical significance was only visible among male students as their T2 score improved to 47, while female students’ score was 46, indicating that there is no statistically significant change (less than the 5th percentile) (see Fig. 3).

The scores have similar characteristics as those of other university students in Japan. The group of female students had a score suggesting that their gender perspective is rather non-traditional compared with that of male students. This finding aligns with the scores of freshmen collected from Hiroshima University (Nishitani, 2020). This observation that female students’ scores tend to be lower than male students is also common among university students from other countries, such as the United States (Iseminger et al., 2020). Among the scores of Japanese male students from Cases 1 and 3, the statistical changes have been evidenced with interventions in the short-term study-abroad program as well as the coursework.

This is an introductory course designed for students who wish to learn about intercultural issues and communication for the first time, and those who are interested in developing careers in the global market in the future. According to the course instructor, it is possible that relatively younger students who are the majority in this class still uphold their ideals of the gender-neutral roles they acquired in high school and from their households. From the instructor’s perspective, as they progress through university life and begin job hunting in the third year, at which time they are introduced to most of the *gendered* industry, it becomes inevitable for them to face the realities of Japanese society, where gender inequality is prevalent. The BEVI score on gender traditionalism at a point in time shows that gender traditionalism among both male and female students is not as low as that of international students, but also not as high as expected, considering the gender gap index in Japanese society. The BEVI scores support the findings of Sakata (2019) that there is no significant gender difference in the leadership experience of Japanese people until their college years. It is highly likely that the gender norms of university students, who have yet to be formally employed or start a career, have not been fixed.

Meanwhile, it is also clear that Case 3 students’ scores show greater tendency toward traditional gender thinking when compared with Case 2 students in the YCCS undergraduate program. While both courses in Cases 2 and 3 deal with globalization, diversity, and other intercultural aspects, the difference in the score is notably significant. As previously explained, in Case 2, the international students’ past experiences may have caused them to think in more liberal and diverse ways with respect to gender traditionalism, as influenced by their own life events and by learning from their peers in the program. Even for local Japanese students, such a transformational learning process can be provided through the efforts of internationalization at universities. As evidenced by research showing that effective educational interventions in short-term study-abroad programs may promote gender understanding (Jessup-Anger, 2008; Squire et al., 2015), active educational interventions may possibly improve college students’ understanding of gender, and short-term study-abroad programs and other forms of intercultural experiences can be effective means of broadening students’ understanding of others.

## Discussion

### Overall findings and potentials for higher education

In comparing the three cases based on the BEVI reports, we observed differences in the scores on gender traditionalism by gender. The three case studies provide essential pieces to the puzzle, and we ought to take them all into consideration for the internationalization of higher education.

Case 1 showed improvement in gender traditionalism among both male and female students after their participation in a short study-abroad program in the Philippines. The findings from Case 1 suggest that a short study-abroad program offers more than learning a new language; its transformative nature aligns with other learning goals, such as gaining a better understanding of others and broadening one’s frame of reference. In Japanese universities, the number of female faculty members at the managerial level remains extremely low (Miyake, 2018), and in companies, the lack of diverse role models for working women is a problem in Japan (Sakata, 2019). Therefore, it is a valuable experience for Japanese students to be exposed to different ways of gender-related division of labor while studying abroad. When a group of students went to the Philippines, the country with the least gender disparity in Asia according to the 2021 Gender Gap Index (World Economic Forum, 2021, pp. 321–322),^3^ they had the opportunity to observe and engage with numerous role models. To maximize the international learning experiences from such a short-term program, it is important that students are well prepared beforehand and also given the opportunity to reflect to enable them to make sense of their growth in understanding others.

Case 2 clearly showed that international students in this group have high levels of openness and flexibility when it comes to gender views. In particular, male students in this course showed the lowest gender traditionalism score among all the cases. Meanwhile, there is a phenomenon across universities that the community of local Japanese students and international students are divided, and the so-called *island* international program has been problematic (Ota, 2016). Universities should act upon their potential to offer opportunities for local and international students to come together to exchange opinions about gender perspectives and discuss solutions to make improvements. There are also international students who are looking for opportunities to interact and learn with Japanese students and vice versa, especially under COVID-19 conditions.

Case 3 consisted of a group of Japanese students and their BEVI scores showed that the gender traditionalism among both male and female students are not as low as in the case of international students but also not as high as expected, considering the gender gap index in Japanese society. Sakata (2019) asserted that there is no significant gender difference in the leadership experience of Japanese students until their college years, and it is highly likely that the gender norms of Japanese university students, who are yet to be formally employed, have not been fixed. During this transitional period, engaging students with diverse and critical perspectives is one of the core responsibilities that universities can undertake. In addition, the data from Cases 1 and 3 showed that gender traditionalism among male students was higher compared to female students.

This study showed that intercultural educational interventions have some potential to improve students’ gender-related views and attitudes, particularly in case of Japanese male students. In this case study, the male students’ gender traditionalism has improved through participating in a short-term study-abroad program (Case 1) and taking an intercultural communication class (Case 3). This indicates that different cultures and diverse opinions could possibly be a trigger for changing their views on gender. According to Pendleton et al. (2016), gender is a relational concept that requires a better understanding of women’s position, but effort must also be made to consider men and masculinity (Pendleton et al., 2016). More importantly, courses on gender should be designed not only for female students but also male students who may be left behind in the discussion on gender inequality.

### Recommendations

This study offers two recommendations for improving gender equality in higher education. First, we propose educational intervention that connects short-term study-abroad programs and gender education by facilitating pre- and post-study sessions, with focus on a wide range of topics, including those that deal with gender, culture, and identity (Wandschneider et al., 2016). It is important to provide systematic learning opportunities by taking a step further and situating short-term study-abroad programs within a regular course. Lutterman-Aguilar and Gingerich (2002) proposed a “problem-posing education” for a study-abroad program where students visit a country that has different attitudes toward gender roles or treatment of women. Students’ frustrations about gender *problems* can be a starting point for their reflection on what they observe and experience in the host country (Lutterman-Aguilar & Gingerich, 2002). Whether seeing gender issues as merely differences or unacceptable phenomena, the role of instructors in the study-abroad programs is crucial in ensuring meaningful learning by students. Lutterman-Aguilar and Gingerich (2002) highlighted that the provision of various reading and video materials and outside speakers to share multiple views on gender encourages students’ reflection in study-abroad programs. Other examples of effective study-abroad programs can be found among those designed for pre-service teachers who have a challenging role to accommodate the growing needs of pupils and their families coming from diverse backgrounds (Vatalaro et al., 2015). Such an international experience is also facilitated by a series of reflections through written journal entries as well as group discussions that have been proven helpful in maximizing students’ learning of intercultural issues.

Second, from the SDG perspective—which explicitly states that universities have an important role promoting sustainability (Leal Filho et al., 2018)—it is important to develop study-abroad programs as well as coursework that interweave SDG themes. A prevalent approach is to use the “Education for Sustainable Development (ESD)” framework^4^, as evidence shows that embedding ESD in educational approaches enhances students’ attitudes toward sustainability (Vare & Scott, 2007). Advocates of ESD call for the promotion of ESD among Japanese universities, and their proposed action plans cover a range of issues including gender equity (Kitamura & Hoshii, 2014). It is also noteworthy that the driving force to develop successful *global human resources* has been one of the predominant rationales of internationalizing higher education in Japan. The prevalence of SDGs has led to the demand for young professionals with knowledge and awareness of sustainability; thus, the work must start in higher education (Liu, 2021). When designing short study-abroad programs or coursework, universities should not leave out aspects of gender that could contribute to liberating norms and assumptions toward a more gender-equal society.

### Limitations of the study

The study made an important contribution by exploring three cases that involved Japanese participants from a short-term study-abroad program as well as international and Japanese students studying at the Japanese university. All three cases in this study had a relatively small number of participants in the program and the courses. The number of participants in university-sponsored, short-term, overseas programs is not large, as seen in the programs in western universities (e.g., Squire et al., 2015, p. 266). Therefore, it is difficult to draw a conclusion to the findings as the entire student population of the Japanese universities is not represented in the study. In addition, the BEVI results showed that there have been statistically significant changes, especially among male students in Case 1 and Case 3; however, this requires a follow-up to reveal factors that have contributed to the positive change. Meanwhile, a comparison of the three cases sheds light on some unexpected positive effects of internationalization efforts in higher education. Such insights can prove useful for improving the program in the future program improvement.

## Conclusions

This study explored the possibility of various opportunities in higher education, such as short study-abroad programs and coursework, to exert positive impacts on university students’ understanding of others, especially regarding gender. The study sheds light on potential interventions for the internationalization of higher education in Japan—not only focusing on language training, but also on setting learning goals to develop broader gender perspectives. A short study-abroad program can be implemented with pre-departure sessions where local Japanese students meet with international students on campus to initiate discussions on global topics, including gender issues. Universities can pay greater attention to providing opportunities for local and international students to come together to exchange opinions about gender perspectives. It is also important that university students be introduced to diverse role models, to help them reconsider their assumptions (Sakata, 2019). The transition from student life to adulthood is a crucial time for university students as they face and become aware of gender inequalities in society. During this transitional period, courses offered on gender should be designed not only for female students but also for male students who may be left behind in the discussion on gender inequality (Pendleton et al., 2016). For future research, it is important to examine the existing learning opportunities by taking a step further to identify attributes that are likely to contribute to the improvement of the gender perspectives. Moreover, it is necessary to investigate at what stage and how college students’ views on gender change from liberal to a more fixed one. By examining the differences in gender traditionalism between freshmen and senior students, it may be possible to develop programs that combine both the intercultural and gender focuses and offer suitable educational interventions at each stage.

## Footnotes

1 The Bevi data were collected by Professor Mitsuyuki Ichimura, the instructor in charge of the class introduced above, “*Gurobaruka to nihonjin*.” The authors obtained permission from professor Ichimura regarding the use of data.

2 It is generally understood that when using the BEVI measurement, there is a significant change when the difference is more than 5 percentile.

3 In the Gender Gap Index (GGI), countries are given a score from zero to one. A score close to one indicates high equality between females and males; on the contrary, a zero score means complete inequality. The Philippines ranked 17 out of 156 countries and scored 0.784.

4 Education for Sustainable Development is a comprehensive learning methodology that encompasses social, economic, and environmental dimensions of formal and informal curriculum.
